# Mechanical protective effect of lens anterior capsule disc on corneal endothelial cells during femtosecond laser-assisted cataract surgery in a rabbit model

**DOI:** 10.1186/s12886-023-02918-0

**Published:** 2023-04-19

**Authors:** Bowen Wu, Xue Ding, Shaowei Li, Dongmei Huo, Fan Zhang, Weiyan Liang, Ling Li, Zexia Dou

**Affiliations:** 1 Aier School of Ophthalmology, Central South University, No.932, Lushan South Road, Changsha, Hunan province China; 2 Beijing Aier-Intech Eye Hospital, Beijing, China; 3 Institute of Corneatology in Aier Eye Hospital, Beijing, China; 4 Tianjin University Aier Eye Hospital, Tianjin, China

**Keywords:** Isolated capsulotomy disc technique, lens anterior capsule disc, femtosecond laser-assisted cataract surgery, Corneal endothelial cell, Phacoemulsification, Ophthalmic viscoelastic devices, Endothelial cell loss

## Abstract

**Purpose:**

To evaluate the effects of a novel technique using an isolated lens anterior capsule disc (LACD) to protect corneal endothelial cells in rabbit eyes during femtosecond laser-assisted cataract surgery.

**Methods:**

Experimental study. 40 rabbits were divided into endothelium-protected (experimental) and control groups, with 20 rabbits in each group. In the experimental group, after femtosecond laser capsulotomy, the isolated capsule disc was lifted to the corneal endothelium by an ophthalmic viscosurgical device. The endothelium was damaged for 1 min with an ultrasonic probe. The control group underwent the same surgery, except that the disc was removed immediately after capsulorhexis. Corneal endothelioscopy was performed preoperatively and on postoperative days (PODs) 3 and 7 to observe endothelial cell counts (ECC) and endothelial cell loss rate. Central corneal thickness (CCT) was measured before and at PODs 1, 3 and 7.

**Results:**

There were 3.59%±1.88% (p < 0.001) and 2.92%±2.14% (p < 0.001) loss of ECC in experimental group at POD3 and POD7, respectively, while those in the control group were 11.62%±7.43% and 10.34%±5.77%, respectively. On POD 1, the difference in central corneal thickness was significant(P = 0.019) between the two groups. At POD 3 and POD 7, CCT was not significantly different (P = 0.597;0.913) between the two groups.

**Conclusions:**

The isolated LACD technique significantly reduced damage to the endothelium caused by ultrasonic energy and protects corneal endothelial cells during phacoemulsification.

## Introduction

Phacoemulsification is the most commonly used technique in cataract surgery. However, phacoemulsification can produce lens fragments, bubbles, free radicals, and especially ultrasound energy, which increases the risk of damaging corneal endothelial cells [1, 2]. Several currently used techniques can be considered for the protection of corneal endothelial cells, including more effective ultrasound, high-quality ophthalmic viscoelastic devices (OVDs), prechop techniques, and femtosecond laser–assisted cataract surgery (FLACS) [3–5]. However, the management of phacoemulsification is made even more challenging in Fuchs’ endothelial dystrophy, previous ocular surgery, and rock-hard cataract patients, who have poor corneal endothelial cell reserves or require more ultrasonic (US) energy during surgery. In these cases, endothelial cell loss is greater, and bullous keratopathy may even occur after uneventful cataract surgery. How to protect corneal endothelial cells more effectively is still one of the most crucial issues for cataract surgeons.

Lens anterior capsule disc (LACD) is a transparent membrane, which is removed during the cataract surgery. For the mechanical protection of the endothelium, we lifted the free LACD, obtained from femtosecond laser–assisted capsulotomy, by OVD. Our group previously introduced this novel endothelium-protected approach called the isolated capsulotomy disc technique [6], which is combined with advanced femtosecond laser-assisted cataract surgery to reduce endothelial cell damage. FLACS was first used in cataract patients in 2009 [7]. The benefits including lower cumulative phacoemulsification time, endothelial cell loss, perfect capsulotomy centration, and the ability to make accurate femtosecond-assisted arcuate keratotomy incisions. Patients with low endothelial cell counts or interested in receiving multifocal intraocular lenses might benefit form FLACS [8]. FLACS is used increasingly in clinical practice in China. There are more than 260 hospitals to practice this procedure now. There are more than 50000 cases of FLACS in China in 2020. In our eye hospital, about 900 FLACS cases every year, which accounting for 30% of whole cataract surgeries (3000 cases/year). During FLACS, we believed that isolated LACDs work as an additional, effective physical protective barrier, which prevents damage caused by US energy, lens fragments, and other damageable factors. This new method offers an extra safety measure during phacoemulsification surgery. However, there were only three clinical cases in our previous pilot study to support this hypothesis. Whether LACD could protect the corneal endothelium from ultrasound damage and whether the direct attachment of LACD could induce damage to the endothelium cells still needs further experiments to verify. Therefore, this study evaluated the effectiveness of this procedure and the possibility of damage to endothelial cells caused by the direct attachment of isolated LACD in rabbit eyes.

## Methods

### Animals

Forty New Zealand white rabbits were acquired from approved vendors and handled according to the Association for Research in Vision and Ophthalmology protocols for animal experimentation in ophthalmology. The study protocol (no. BJAEYZ201804A01) was approved by the Medical Ethics Committee of Beijing Aier-Intech Eye Hospital and followed the guidelines for animal ethics. All animals were healthy and free of ocular disease.

The right eyes of forty New Zealand white adult rabbits weighing 2.5 to 3.0 kg were divided into two groups, with 20 rabbits in each group. One was the experimental group (LACD group), which was treated with an isolated capsulotomy disc, LACD, to protect corneal endothelial cells from US energy, and the other was the control group (conventional FLACS group), which received regular FLACS without the novel endothelium protection procedure.

Topical levofloxacin (Santen Pharmaceutical, Inc. Japan) was used on the operative eye 4 times a day 3 days before surgery. Six drops of topical pharmacologic dilation (tropicamide 0.5% and phenylephrine 10% eyedrops, Santen Pharmaceutical, Inc. Japan) were administered one hour before surgery. All surgeries were performed by the same experienced surgeon (S.W.L.). Anesthesia was administered by intravenous injection of pentobarbital sodium according to animal weight (100 mg/kg), and 0.5% proparacaine eye drops (Alcon Laboratories, Inc.USA) were applied topically.

**Fig. 1:**
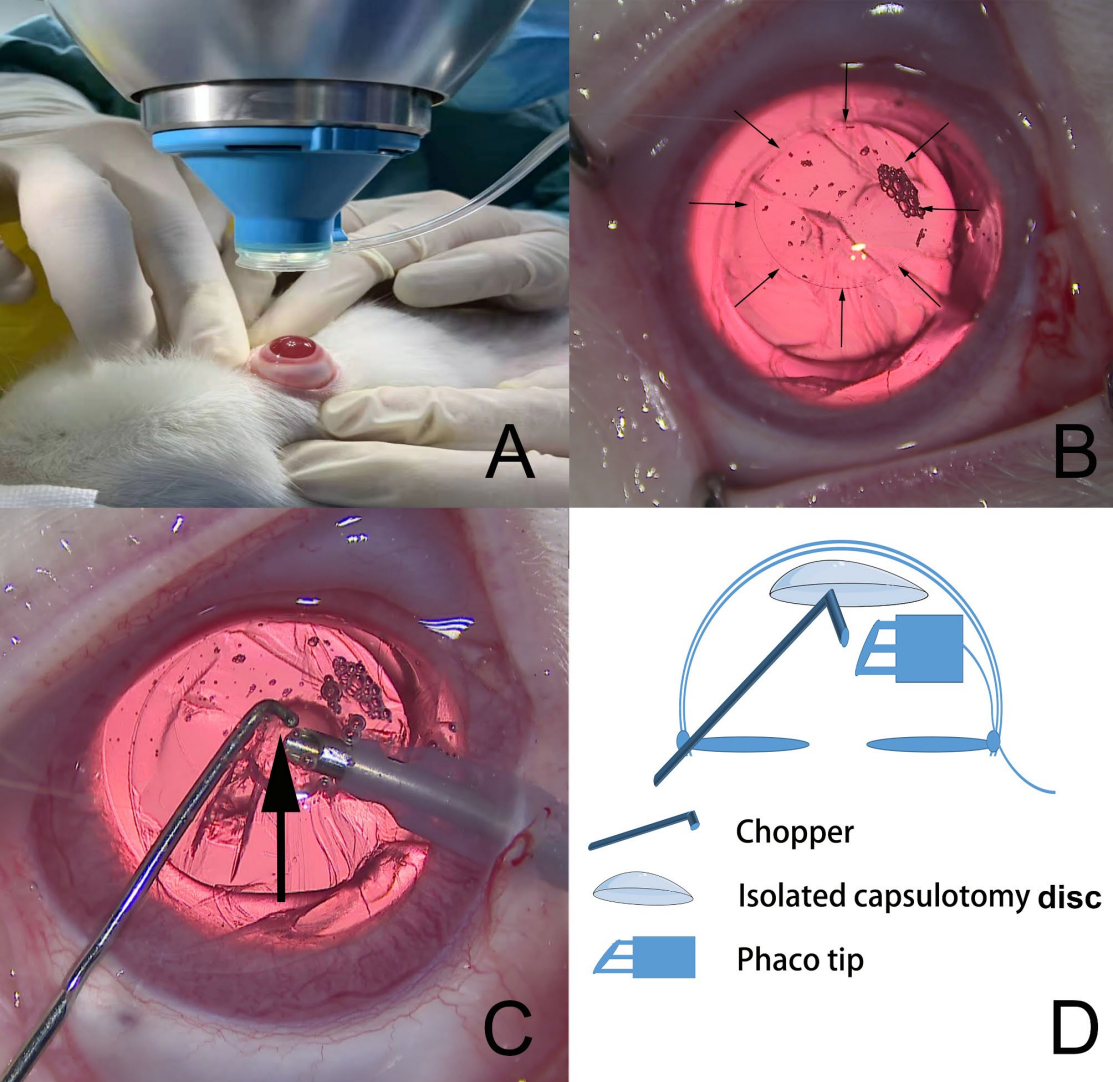
Surgical procedure of the isolated lens anterior capsule disc technique. **A**. After peribulbar injection of 0.9% normal saline, more of the eyeball was exposed, and the PI was more easily contacted. **B**. Using OVD to lift the disc to the endothelium. Thin arrow surrounding the isolated capsule disc. **C**. Insertion of the phacoemulsification tip under the mechanical protector and positioning of the bevel toward the central corneal endothelium (upward). The side-port incision was created at 2 o’clock. A chopper hook bend was used as the reference object to ensure that the phaco tip was always positioned 1.5-2.0 mm to the endothelium. The bold arrow shows the relative position of the chopper hook and phaco tip. **D.** Schematic illustration of the isolated capsulotomy disc technique

To prevent the rabbit’s third eyelid from obstructing the contact between the eyeball and patient interface (PI) and to perform a successful capsulotomy, all surgical eyes initially received a peribulbar injection of 2 to 3 ml of 0.9% normal saline, causing obvious protrusion and exposing more of the eyeball (Fig. 1A). The procedure for standard laser capsulotomy was used for both groups (LenSx, Alcon Surgical, Inc., USA). In addition, the following capsulotomy specifications were used: the diameter was 6 mm; the capsulotomy incision depth was 700 μm, 350 μm above and 350 μm beneath the anterior lens capsule; the energy was 4 µJ; the spot separation was 4 μm; and the layer separation was 3 μm.

### Experimental phacoemulsification group (video 1)

The phacoemulsification procedures were performed under an operating microscope in an aseptic manner. A wire lid speculum was used to open the eyelids. A main sclerocorneal incision of 3 mm was created at the 10 o’clock position, and 0.5 mL of heparin (2500 USP units/mL) was injected into the anterior chamber immediately to control anterior chamber coagulation. OVD (DisCoVisc, hyaluronic acid 1.6%–chondroitin sulfate 4.0%, Alcon Laboratories, Inc. USA) was injected under the LACD and pushed it up to the endothelium, ensuring that the anterior chamber was fully filled with the OVD (Fig. 1B). The side-port incision was created at the 2 o’clock limbus. The chopper was introduced through the side-port incision into the anterior chamber. As a reference object, the chopper tip was positioned adjacent to the endothelium, and then the phaco tip was placed at the position of the chopper hook bend, which was 1.5-2.0 mm from the endothelium, facing the endothelium and adjacent to the lens (Fig. 1C and D). The constant longitudinal phacoemulsification power was set to 80%, and 10 s of alternate intermittent phacoemulsification was applied for a total elapsed time of 1 min of US exposure (total time of 2 min) (Fig. 1B and C). The rates of irrigation/aspiration (I/A) and vacuum were set at 12 cc/min and 300 mmHg, respectively (Infiniti, Alcon Surgical, Inc., USA). After ultrasound injury, the lens material was cleanly aspirated, preserving the capsule. If the attached disc separated from the endothelium during the damage process, the eye was excluded from the study.

Then, the phacoemulsification parameter was reset. Torsional, continuous phacoemulsification power was set to 50%, the rate of aspiration was set at 40 cc/min, and the vacuum was set to 400 mmHg. An I/A tip was used to remove the remaining disc, OVD, and cortical material as in regular phacoemulsification surgery.

### Control phacoemulsification group

In the control group, the operative eyes were treated in the same manner as in the experimental group except that the isolated disc was removed once the main corneal incision was created.

### Clinical examinations

Detailed clinical examinations were performed by the same masked ophthalmologist (B.W.W.). A slit-lamp examination (LS-6 Slit Lamp, Shangbang, China) was performed to check corneal edema and intraocular inflammation preoperatively and at POD 1, POD 3 and POD 7. Anterior segment optical coherence tomography (RTVue XR, Optovue, USA) was performed to observe the central corneal thickness (CCT) preoperatively and at POD 1, POD 3 and POD 7. The in vivo endothelial cell count (ECC) was acquired using specular microscopy (CEM-530, Nidek, Japan) preoperatively and at POD 3 and POD 7. Topical tobramycin/dexamethasone (Alcon Laboratories, Inc. USA) was used in the surgical eyes 4 times a day until 7 days after surgery. All rabbits were humanely sacrificed by intravenous injection of a pentobarbital sodium overdose.

### Statistical analysis

SPSS 22.0 software (IBM, Armonk, NY, USA) was used to analyze the means and standard deviations of ECC, CCT and ECL. The independent samples t test was used to compare differences in the parameters between the 2 groups. The level of statistical significance was defined as a P value less than 0.05.

## Results

**Fig. 2:**
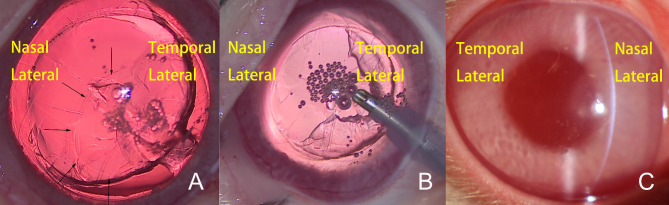
Decentered attached disc. **(A)** The isolated disc attached to the corneal temporal lateral. Thin arrow surrounding the isolated capsule disc. **(B)** A phaco tip was placed at the central cornea and damaged endothelial cells. **(C)** Corneal nasal focal edema at POD 1

Decentered attached discs, the capsular discs were well separated from the lens but later becoming decentered when adhering to the endothelium, were noticed in two eyes (Fig. 2A and B) in the experimental group. The other eyes experienced no adverse events. In the control group, all surgical procedures were uneventful.

**Fig. 3:**
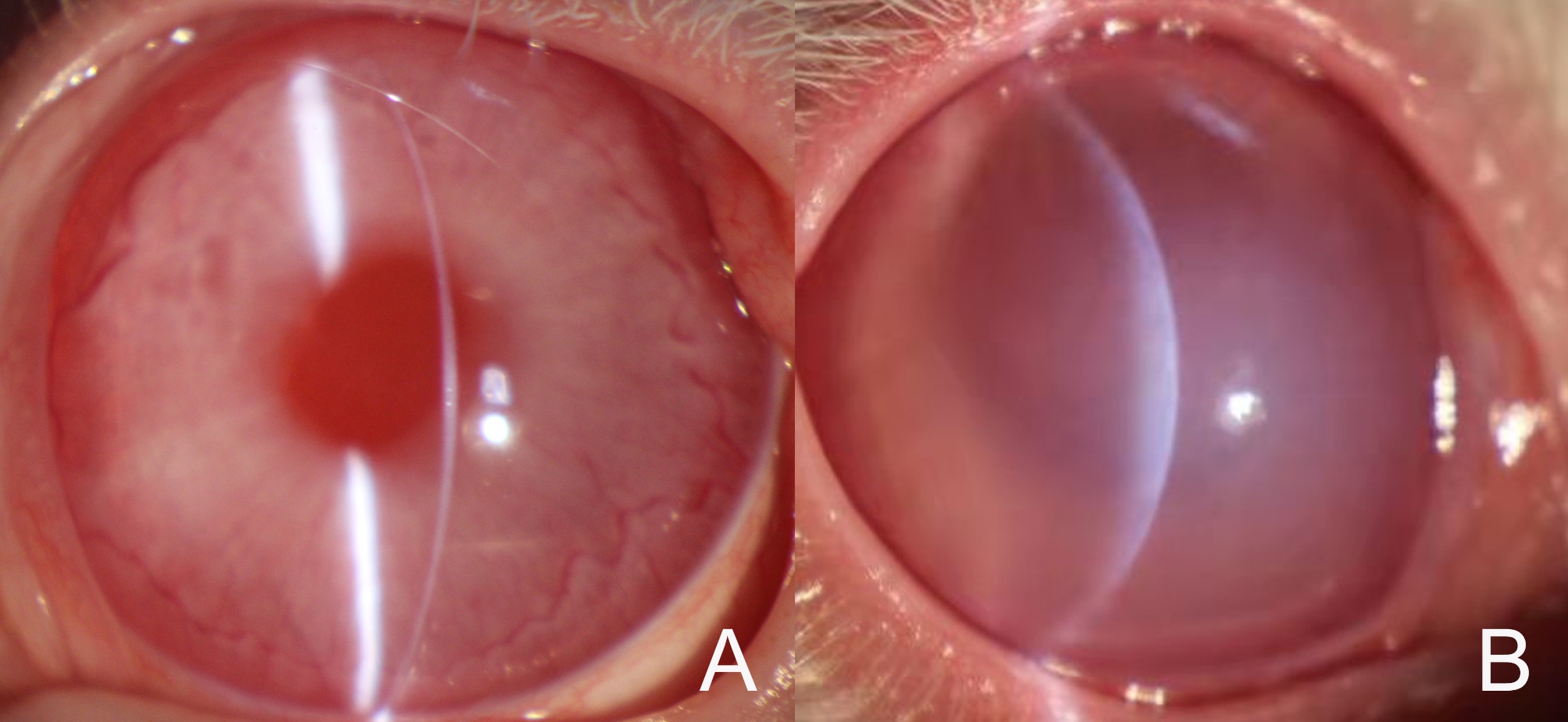
Slit lamp photography of two groups. **A**. The cornea was clear in the experimental group at POD 1. **B**. Seriously diffuse edema in the control group at POD 1

In the experimental group, focal corneal edema was observed in two decentered disc cases one day postoperatively (Fig. 2C). Corneal edema resolved 2 and 3 days after surgery. The other eyes were clear after surgery (Fig. 3A). In the control group, 9 eyes had diffused edema, 11 eyes had focal edema, and the edema resolved 3 to 5 days after surgery (Fig. 3B).

The control group had more ECC loss than the LACD group (Table 1). Preoperatively, the mean ECC was not significantly different between the two groups. Postoperatively, the ECC was decreased in both groups. The decrease was significantly greater in the control group than in the experimental group (P < 0.001).

**Table 1:** Preoperative and postoperative ECCs ($\bar x \pm s$)

Parameter	Endothelial Cell Count (cells/mm^2^)			Endothelial Cell Loss (%)	
	Preop	3 day postop	7 day postop	3 day postop	7 day postop
Experimental Group	2812 ± 127	2712 ± 126	2730 ± 135	3.59%±1.88%	2.92%±2.14%
Control Group	2744 ± 142	2421 ± 195	2457 ± 159	11.62%±7.43%	10.34%±5.77%
*P Value*	0.117	<0.001	<0.001	< 0.001	<0.001

Table 2 shows the changes in CCT. At day 1 postoperatively, the CCT was thicker in the control group than in the experimental group, and the difference was statistically significant (P = 0.019). There was no significant difference in corneal thickness (p > 0.005) between the 2 groups 3 days and 7 days postoperatively.

**Table 2:** Preoperative and postoperative CCT ($\bar x \pm s$)

Parameter	central corneal thickness(µm)			
	Preop	1 day postop	3 day postop	7 day postop
Experimental group	337 ± 23	372 ± 40	352 ± 29	344 ± 23
Control group	328 ± 22	434 ± 106	357 ± 26	345 ± 33
*P Value*	0.204	0.019 *	0.597	0.913

* *P* < 0.05

## Discussion

The main cause of corneal endothelium damage during phacoemulsification is direct injury from ultrasound energy [9]. Therefore, we created a rabbit model of endothelial cell damage by phaco ultrasonic energy. That is, the high frequency of ultrasonic energy was transferred to the corneal endothelium directly by the phaco tip toward and 1.5-2.0 mm from the endothelium, and the average anterior chamber depth of the adult New Zealand rabbit eye is about 2.08 mm [10]. To establish a model of direct damage to endothelial cells by ultrasound, we used traditional ultrasound in this experiment rather than twisting ultrasound commonly used in clinical practice. We just want to build a model of ultrasound energy damage to the corneal endothelium. Referring to previous study [11], 70% longitudinal ultrasound energy was used to damage the endothelium. However, in pre-experiments, we found that 2.5 min was too long. We increased the energy to 80% and reduced time to 1 min. It not exactly simulated the clinical phacoemulsification method and parameters. The clinical approach alone was difficult to produce damage to the endothelium for a short period of time. The animal models were used to induce maximum endothelial cell damage and to clearly validate the protective effect of this method on endothelial cells. The results showed that this animal model induced significant corneal endothelium damage, which presented as severe corneal edema and a high endothelial cell loss rate in the control group.

New Zealand white rabbits were selected as experimental animals in this experiment. Because corneal parameters such as endothelial cell density, central corneal thickness, and corneal diameter decrease with age were similar to humans. New Zealand white rabbits are the most commonly used experimental animal models for corneal research [11]. Observation on the first day after cataract surgery was important to the clinician. The cornea should be clear at POD1 usually, and if there was edema, it was evidence of endothelial damage. Mild damage might be repaired at POD 3. And observation at POD1 might reduce the impact of endothelial cell proliferation on the outcome. Although the corneal endothelial cells remain the proliferative ability after injury, adult New Zealand white rabbits usually don’t initiate endothelial cell proliferation until 72 h to 1 week after injury [10, 11]. Therefore, we chose 1 day, 3 days and 7 days after surgery as observation points.

The protective effect of the LACD was significant in the experimental group. The corneal endothelial cell loss rates in the experimental group were 3.59%±1.88% and 2.92%±2.14% at POD 3 and POD 7, respectively, while those in the control group were 11.62%±7.43% and 10.34%±5.77%, respectively. The difference was statistically significant, indicating that an isolated LACD has a significant protective effect on corneal endothelial cells. According to the literature, the average endothelial cells lost is between 100 cells/mm^2^ and 400 cells/mm^2^ during cataract surgery [12, 13], In this study, the number of endothelial cells lost in the control group was about 323 cells/mm2 at POD3. However, this experiment does not fully simulate the cataract surgery process. In the experimental group, we could not keep all the LACDs on-site during phachoemulsification. This is mostly because of the difference in anatomy between human and rabbit eyes. We found marked fibrous exudates formed in the rabbit’s anterior chamber after femtosecond laser treatment, which reduced the period of attachment time. Considering that the aim of this study was to evaluate the protective effects of the disc, not to optimize the procedure, we only enrolled rabbits with discs attached long enough in the experimental group. It was meant the LACD apposition over the course of 6 ultrasounds for a total of 1 min. If it fell off before the ultrasound was completed, it was excluded. To make the disc attach stable, we injected heparin into the anterior chamber before surgery and adjusted the phacoemulsification parameters, that is, we set the I/A flow rate to 12 cc/min and the vacuum to 300 mmHg. Although the changed parameters were far from clinical use, it is still an ideal animal model to prove the effectiveness of discs.

Two eyes showed focal corneal edema on the first day after surgery in the experimental group. This was related to the decentered attachment of the discs during surgery, which was confirmed by reviewing surgical videotapes after surgery (Fig. 2A). Because the phaco tip was placed toward the central part of the cornea to produce endothelial cell damage (Fig. 2B), the uncovered central area of the cornea was edematous, and the paracentral part covered by discs was clear at POD 1 (Fig. 2C). This finding confirmed the fact that the disc played a strong protective role.

Theoretically, an OVD may also protect the corneal endothelium during phacoemulsification [14]. However, the OVDs were almost completely removed from the anterior chamber halfway through the phacoemulsification process. Moreover, even if the residual OVD produced a certain degree of protection, under the same US energy damage conditions, the postoperative corneal endothelial cell loss rate in the control group was much higher than that in the experimental group. Therefore, we can conclude that the isolated LACD plays a decisive role in protecting corneal endothelial cells. LACD is a smooth basement membrane and obtained from the same patient minimizing the risk of the biotoxicity. In our study, LACD act as a mechanical barrier to protect the corneal endothelium. Additionally, the minimal endothelial cell loss rate in the experimental group indicated that direct contact between the disc and corneal endothelium caused almost no harm to endothelial cells.

If we want the disc to remain attached to the corneal endothelium during phacoemulsification, the following skills are key to success. Firstly, the discs were stable when low-viscosity dispersive OVDs rather than higher molecular weight cohesive OVDs were used during phacoemulsification [11, 14]. This is because the dispersive OVDs tend to be aspirated out gradually, and the disc will not be drawn out with the OVDs during phaco and I/A procedures. Secondly, the OVDs pushes the disc against the corneal endothelial surface expelling the interposed aqueous humor, resulting in a siphoning effect that making the disc adhering to the corneal endothelial layer. However, if the anterior chamber pressure is not high enough, the aqueous humor between the layers cannot be completely discharged and the capsule disc cannot be tightly adhered. The OVDs injected must be enough to raise the anterior chamber pressure to about 30–40 mmHg to make the LACD tightly adhered, which is similar to the principle of Descemet membrane endothelial keratoplasty surgery. disc In our experiments, we noticed that at the late stage of phacoemulsification and during the I/A procedure, the margin of the attached disc began to wrinkle and curl due to the turbulence of the fluidic system. This breaks the sealed chamber between the disc and endothelial layer and leads to the disc detaching from the endothelium at the end of surgery. In this study, there was no case in which the LACD could not be detached at the end of the operation due to the tight attachment.

There are four major limitations to our study. First, we chose the rabbit eye as a surgical model. Unlike the human endothelium, rabbit corneal endothelial cells have high mitotic activity. After 2 to 3 weeks, the rabbit cornea recovered to normal transparency. This hampered the long-term observation in this experimental study. Second, rabbit lenses are soft, so they cannot simulate the surgical process of actual dense nuclei, and whether the disc can protect endothelial cells from nuclear fragments is still unknown. Third, the surgical progress was good in the rabbit eye, but it was highly different from clinical situations. Further study is needed before this procedure can be applied to the clinic. And there are still many challenges in clinical application, for example, how to adjust the rate and vacuum of the fluid flow to ensure effective ultrasound and keep the capsule disc from detached easily, finding the appropriate pressure for proper apposition time and easy detachment from the endothelial surface at the end of ultrasound, training a cataract surgeon in surgical techniques like corneal endothelial transplantation, etc. Finally, we did not analyze the difference between experimental and control groups by histology and immunohistochemistry findings. These studies may provide more meaningful information regarding the possible mechanism of the protective role of the disc protection technique. However, the main purpose of our study is to prove the feasibility of a novel protective technique, and further analysis would be hopefully carried out in our next research trial.

In summary, our experimental results prove that the LACD protection technique has a protective effect on the corneal endothelium and provides an extra measure of endothelium safety during phacoemulsification. However, further understanding regarding appropriate surgical parameters, such as flow rate and vacuum, is required so that the disc protection technique can eventually be used clinically in humans. Meanwhile, in clinical femtosecond laser capsulotomy, the diameter of the anterior capsule disc is only approximately 5 to 5.5 mm, which protects a limited region of the endothelium. Therefore, surgeons still need to minimize surgical trauma by applying all traditional methods, even though the isolated disc technique can protect endothelial cells somewhat from phacoemulsification.

## Data Availability

The datasets used and/or analyzed during the current study are available from the corresponding author on reasonable request.

## Abbreviations

LACD: lens anterior capsule disc
POD: postoperative day
ECC: endothelial cell counts
ECL: endothelial cell loss rate
CCT: central corneal thickness
OVD: ophthalmic viscoelastic device
FLACS: femtosecond laser–assisted cataract surgery
US: ultrasonic
PI: patient interface
I/A: irrigation/aspiration.
